# Global Genetic Diversity of Human Metapneumovirus Fusion Gene

**DOI:** 10.3201/eid1006.031097

**Published:** 2004-06

**Authors:** Guy Boivin, Ian Mackay, Theo P. Sloots, Shabir Madhi, François Freymuth, Dana Wolf, Yonat Shemer-Avni, Herbert Ludewick, Gregory C. Gray, Éric LeBlanc

**Affiliations:** * Centre Hospitalier Universitaire de Québec and Laval University, Québec City, Canada;; † Royal Children’s Hospital, Herston, Queensland, Australia;; ‡ University of Witwatersrand, Bertsham, South Africa;; § Centre Hospitalier Universitaire de Caen, Caen, France;; ¶ Hadassah University Hospital, Jerusalem, Israel;; # Soroka Medical Center, Beer Sheva, Israel;; ** University of Iowa, Iowa City, Iowa, USA

**Keywords:** Human metapneumovirus, fusion gene, phylogenetic analysis, sequencing, diversity, genotypes

## Abstract

We analyzed 64 human metapneumovirus strains from eight countries. Phylogenetic analysis identified two groups (A and B, amino acid identity 93%–96%) and four subgroups. Although group A strains predominated, accounting for 69% of all strains, as many B as A strains were found in persons >3 years of age.

Studies from various parts of the world have identified human metapneumovirus (HMPV) as one of the leading causes of hospitalization for acute respiratory tract infections in young children (*1**–**5*). Severe respiratory infections associated with HMPV have also been reported in elderly and immunocompromised persons (*6**,**7*). Studies from our group (*6**,**8*) and others (*2**,**5*) have identified two major lineages of HMPV, with some studies indicating the splitting of those groups into subgroups. Recently, the complete genomic sequence of a representative strain from each of the two groups was determined (*9*). In this study, our objective was to analyze the fusion (F) gene sequences of a large set of HMPV strains collected from various countries over several years and to identify sequence signatures in different HMPV subtypes.

## The Study

HMPV sequences included were from isolates grown in LLC-MK2 (monkey kidney) cells or polymerase chain reaction (PCR)-amplified products from nasopharyngeal aspirates (NPA) (*1**,**3**,**5**,**6**,**10*). Viral RNA was extracted from 200 µL of cell culture supernatants or NPA specimens by using the QIAamp viral RNA Mini Kit (QIAGEN, Inc., Mississauga, ON, Canada). For phylogenetic studies, nucleotide sequences were obtained from amplified HMPV F-gene products as previously described (*6*). The sequence region comprising nucleotides 60-708 of the F gene was entered into a multiple alignment generated by Clustal-W and corrected through final visual inspection with the SeqLab application (Wisconsin package version 10.3, Accelrys Inc., San Diego, CA). Phylogenetic analysis was performed by using distance methods with the PAUP 4.0b10 program (Sinauer Associates, Inc., Sunderland, MA) for the Macintosh. The parameters for the distance method were Kimura 2-parameters, using the neighbor-joining algorithm. Five hundred additional bootstrap analyses were performed on those phylogenetic trees.

**Figure 1:**
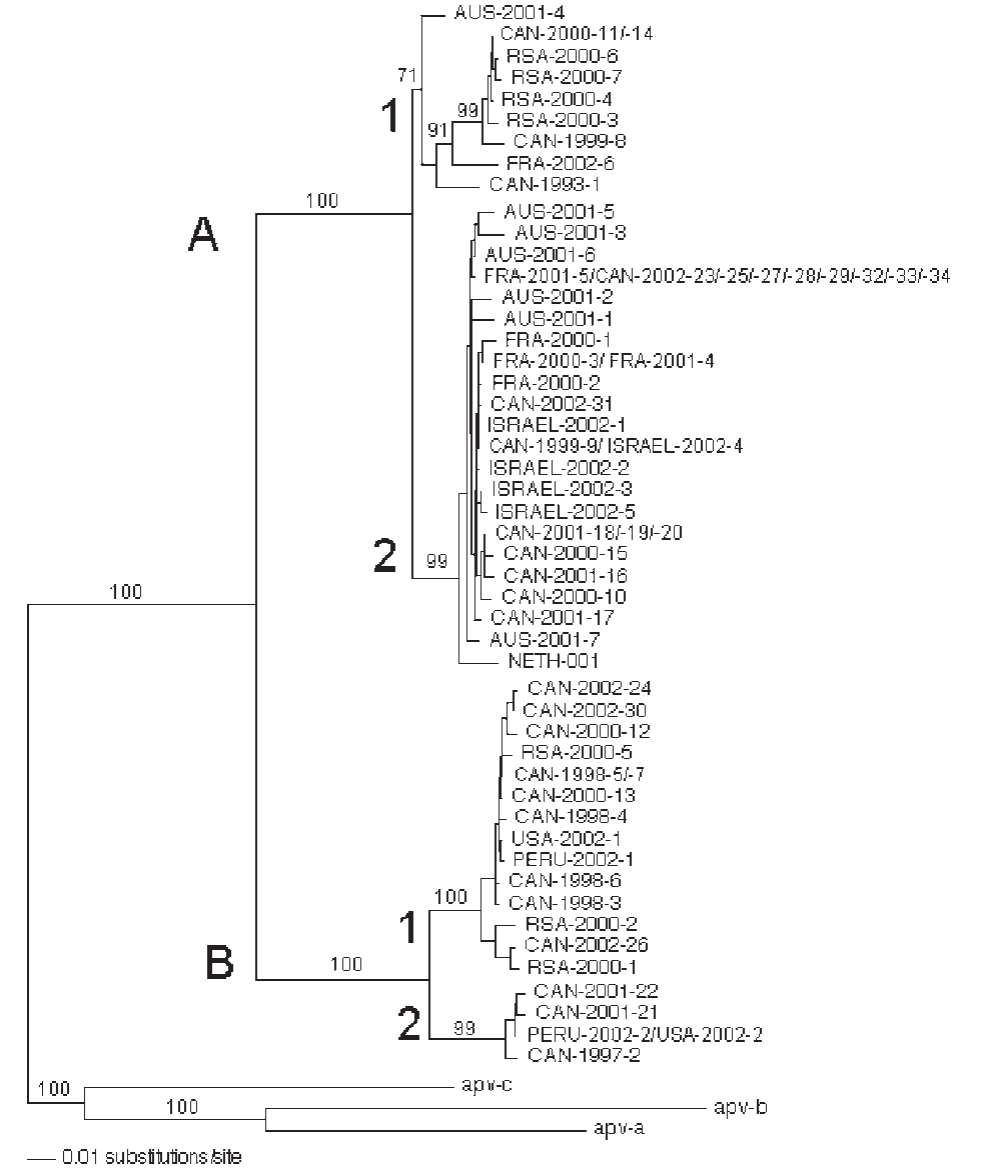
Phylogenetic analysis of the fusion (F) gene of 64 human metapneumovirus (HMPV) strains recovered from various countries (CAN, Canada; RSA, Republic of South Africa; FRA, France; AUS, Australia; NETH, the Netherlands). Neighbor-Joining consensus tree was obtained from the nucleic acid alignment representing nucleotides 60–708 of the HMPV prototype sequence NETH-001. Numbers represent the frequency of occurrence of nodes in 500 bootstrap replicas.

A total of 64 HMPV sequences were analyzed, 34 from Canada (years 1993–2002) and 30 from various other countries (years 2000–2002): Peru (n = 2), the United States (n = 2), France (n = 6), Israel (n = 5), Republic of South Africa (n = 7), Australia (n = 7), and the prototype strain 001 (GenBank accession no. AF371337) from the Netherlands. Phylogenetic analysis of the HMPV F gene showed the existence of two main groups (A and B) that could be further subdivided into two subgroups (1 and 2; Figure 1). Bootstrap analysis strongly supported dividing HMPV into the A and B clusters (100% of bootstrap replicas) as well as subdividing them into B1, B2, and A2 (99%–100% of bootstrap replicas). However, the topology of the tree regarding the A1 subgroup was supported by only 71% of the bootstrap replicas. This result may be explained by the strain AUS-2001-4, which could not be firmly assigned to any of the two A subgroups. In addition, even though the strain CAN-1993-1 was strongly supported as a member of the A1 group by 91% of the bootstrap replicas, it was clearly the most divergent, as can be expected from an older strain.

Identical F-gene sequences (648 nt) were found for some strains isolated in the same year from the same country (CAN-1998-5/7, CAN-2000-11/14, CAN-2001-18/19/20) but also for strains isolated in different years from different countries (ISRAEL-2002-4 and CAN-1999-9, FRA-2001-5 and CAN-2002-23/25/27/28/29/32/33/34). Topology of the phylogenetic tree was also supported by analysis of homology between sequences. Nucleotide identity between groups A and B was 81.5%–85.3%, whereas it was 91.6%–95.3% and 92.0%–94.1% between subgroups A1–A2 and B1–B2, respectively. The sequences within the three subgroups A2, B1, and B2 shared a nucleotide identity of 96.3%–99.0%, 96.0%–99.9%, and 97.2%–99.4%, respectively. The A1 group was the most divergent; sequences shared 94.4%–99.9% of nucleotide identity, which was consistent with phylogenetic data. AUS-2001-4 and CAN-1993-1 strains shared only 94.2% and 96.5% of nucleotide identity with other members of the A1 subgroup. At the amino acid level, the identity was 93.1%–96.3% between groups A and B, whereas it was 96.3%–99.1% and 98.1%–99.1% between subgroups A1–A2 and B1–B2, respectively. Within subgroups, amino acid identity was 98.2%–100%, 96.8%–100%, 99.5%–100%, and 99.1%–100% for subgroups A1, A2, B1, and B2, respectively. Based on phylogenetic analysis, the distance between the two HMPV groups was slightly smaller than between prototypes from the two respiratory syncytial virus (RSV) groups (14.5 versus 16.5 substitutions/100 residues, data not shown).

**Figure 2:**
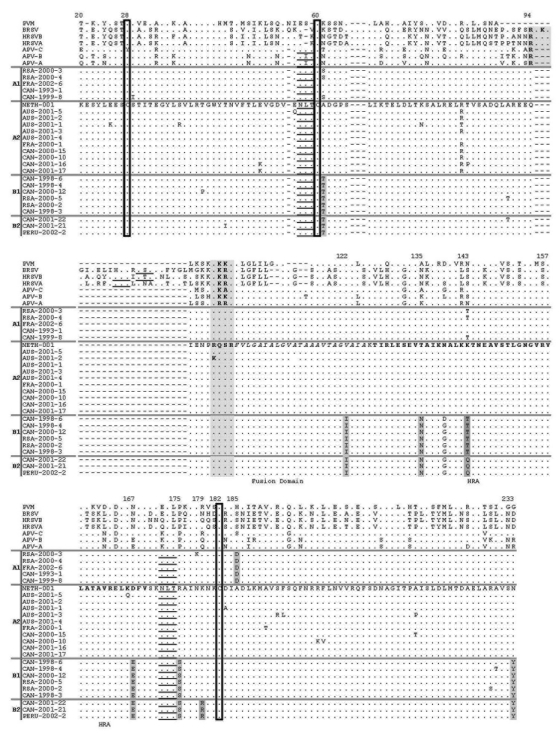
Amino acid sequence alignment of the fusion (F) protein of various human metapneumovirus (HMPV) strains and other paramyxoviruses. Amino acid numbering is based on the sequence of the HMPV strain NETH-001 (GenBank accession no. AF371337). Amino acids shown are those different than NETH-001. Boxed residues represent conserved cysteines. Potential N-glycosylation sites are underlined. The fusion domain is indicated by italics in the consensus sequence, whereas the heptad repeat A region is indicated by bold characters. Shaded residues represent significant substitutions between HMPV groups and subgroups. Note that only distinct HMPV strains were included in the alignment. PVM, pneumonia virus of mice; BRSV, bovine respiratory syncytial virus; HRSV, human respiratory syncytial virus; APV, avian pneumovirus; RSA, Republic of South Africa; CAN, Canada; FRA, France; AUS, Australia; NETH, the Netherlands.

An amino acid alignment of all distinct HMPV F sequences (representing amino acids 20–233 of the prototype strain NETH-001), as well as those of other metapneumoviruses and pneumoviruses, is shown in Figure 2. Cysteine residues were conserved at positions 28, 60, and 182 in all HMPV strains. Analysis of this multiple sequence alignment showed six amino acid substitutions found at positions 61, 122, 135, 167, 175, and 233 that may be used as signature sequences to differentiate group A and B strains. In addition, we also identified an amino acid substitution at position 185 that may allow us to differentiate the A1 subtype from others, whereas substitutions specific to B2 subtypes were located at positions 143 and 179. Some of these substitutions were located in potential functional domains, such as the fusion domain (codon 122) and the heptad repeat A (HRA) region (codons 135, 143, and 167). Two potential N-glycosylation sites were found in all HMPV sequences.

We investigated associations between HMPV genotypes and demographic or clinical data, although the small number of non-Canadian strains limited analyses. Based on phylogenetic analysis, a total of 44 (68.8%) HMPV strains were classified in group A (9 [14.1%] A1 and 35 [54.7%] A2), whereas 20 (31.2%) belonged to group B (15 [23.4%] B1 and 5 [7.8%] B2). Group B strains accounted for 38.2% and 23.3% of Canadian and non-Canadian strains, respectively (p = 0.20). No B strains were recovered from France, Australia, and Israel as part of this study. Co-circulation of both A and B strains was found in Canada during 2000, 2001, and 2002 as well as in South Africa during 2000. Among the 26 HMPV strains recovered before 2001, 57.7% were from group A compared to 42.3% from group B, whereas the ratios were 76.3% and 23.7% for the 38 strains analyzed in 2001 and 2002 (p = 0.12). When analysis was restricted to Canadian strains, group A strains accounted for 46.7% of all HMPV recovered before 2001 and 73.7% of HMPV recovered after 2001 (p = 0.11). All but one of the recent (year 2001 or later) A strains were from the A2 subgroup.

Subsequent analyses were performed without the prototype strain NETH-001, for which no detailed information was available. Most strains from the Northern Hemisphere (41 [87.2%] of 47) and the Southern Hemisphere (11 [68.8%] of 16) were recovered over the typical respiratory virus season, spanning a 5-month period during winter and spring. Most (69.8%) of the HMPV strains were recovered from young children <3 years, whereas only 25.6% were from adults >18 years of age, although this finding may only reflect more intense investigation into viral cases in children. Group B strains accounted for 25% of total HMPV strains from study participants <3 years of age compared to 47.4% of those from patients >3 years of age (p = 0.08). The ratios of group A/group B strains were 62.5%/37.5% for the 32 male and 74.2%/25.8% for the 31 female patients (p = 0.32). Groups A and B strains were associated with a similar proportion of cases of pneumonitis (12 [27.9%] of 43 vs. 7 [35%] of 20, p = 0.57). Finally, although group B strains were found in approximately half of the positive LLC-MK2 cultures (14 [53.8%] of 26), such strains only represented 16.2% of total HMPV strains detected by reverse transcription–PCR from NPA samples (p = 0.002).

## Conclusions

Our results, based on a large dataset of viral strains collected over several years from the Northern and Southern Hemispheres, confirm two main HMPV groups and at least four minor subgroups. One strain (AUS-2001-4) could not be ascribed to one of the two A subtypes and may constitute a third subtype. We have further demonstrated that both HMPV genotypes could circulate in a single year during a typical respiratory virus season, i.e., over the winter and early spring months in countries in both hemispheres. Our phylogenetic data were based on analysis of the HMPV F protein, one of two viral glycoproteins considered the major antigenic determinant in RSV (*11*). Our data confirm that HMPV is more closely related to avian pneumovirus type C than to RSV, which is classified in a separate genus (Pneumovirus) within the same subfamily.

Overall, the amino acid sequence identity of the two HMPV F groups was slightly higher than that calculated for the two RSV groups (93%–96% vs. 89%) (*11*). Although only the first half of the F gene was sequenced in this study, our data indicate that all A and B strains can be easily differentiated on the basis of unique amino acid changes, some of which are located in functional regions of the protein, e.g., the putative fusion domain and the HRA region implicated in paramyxovirus fusion to their cell receptors. Some of the latter amino acid changes were nonconservative. Of note, the sequence signatures reported here were also present in two other recent Canadian isolates representing the two major HMPV groups (*12*). Additional studies will be required to evaluate the impact of such changes on pathogenesis and immune response.

Close to 70% of all HMPV strains belonged to group A with a possible shift towards more A2 strains in recent years. However, more young children were evaluated in our study, and the group A genotype infects three times as many children <3 years of age. In addition, group B strains, which occurred more frequently in adults, could have been underestimated, as suggested by the detection of fewer group B strains in clinical samples (16%) than in infected LLC-MK2 cells (54%). In that regard, most HMPV PCR primers designed so far have been selected from the sequence of strain NETH-001 (which belongs to group A2 in this study), and consequently, they may not have been optimal to detect group B strains. Alternatively, isolating HMPV group A strains may be less efficient in LLC-MK2 cells. Additional studies are required to validate these hypotheses. In RSV, group A strains are often thought to be associated with more severe disease than are group B strains (*13*). However, we found no differences in severity between the two HMPV genotypes when we used pneumonitis as the clinical endpoint in this small, retrospective study.

Our study has some limitations. We analyzed relatively few non-Canadian strains over a short period of time, consistent with the recent description of this viral pathogen in 2001. Also, much more genetic variability could have been observed by sequencing the gene encoding for the attachment glycoprotein (G gene) as suggested by RSV and limited HMPV sequences (*14**,**15*). Using different PCR primers to initially identify HMPV and studying different populations in the various centers may have also introduced bias in interpreting our results. Nevertheless, our study confirms the worldwide distribution of HMPV and provides initial insights into the epidemiology of the two main viral genotypes.
